# Changes in pain, quality of life, sleep, and mental health after uncomplicated spinal neurosurgery in palestine: a prospective study of patient-reported outcomes

**DOI:** 10.1038/s41598-025-25850-3

**Published:** 2025-11-21

**Authors:** Ahmad Abdulrahman Daqqa, Jamal Qaddumi, Basma Salameh, Ahmad Hanani

**Affiliations:** 1 Palestinian Medical Complex Hospital, Palestinian Ministry of Health, Ramallah, Palestine; 2 Faculty of Nursing, An Najah National University, Nablus, Palestine; 3 Department of Nursing, Faculty of Nursing, Arab American University, Jenin, Palestine; 4 Department of Public Health, Faculty of Medicine and Health Sciences, An- Najah National University, Nablus, Palestine

**Keywords:** Patient-reported outcomes measures, Spinal neurosurgery, Pain, Quality of life, Sleep quality, Sleep disturbance, Mental health, Diseases, Health care, Medical research, Neurology, Neuroscience

## Abstract

The use of patient-reported outcomes measures (PROMs) is an application of the volume-to-value-based healthcare services, and was quantitatively used in the field of neurosurgery. Therefore, the current study aimed to investigate the preoperative and early postoperative, as well as changes and factors of changes, in specific PROMs among spinal neurosurgery patients in a tertiary hospital in Palestine. The study was conducted using a prospective longitudinal design on a convenience sample of 99 lumbar and 35 cervical spine neurosurgery patients, who were interviewed to fill in a preoperative and one-month postoperative questionnaire that measures pain, quality of life (QoL), sleep quality, and mental health PROMs. Valid versions of Arabic translated tools were used, including Neck Disability Index (NDI), Oswestry Disability Index (ODI), EuroQoL (EQ-5D-5 L), Pittsburgh Sleep Quality Index (PSQI), Epworth Sleepiness Scale (ESS), and Patient Health Questionnaire (PHQ-9). Data were analyzed using SPSS with full commitment to ethical considerations of anonymity and confidentiality. The patients had a mean age of 49.16 years, and were 50.7% females, 74.6% married, 59.7% underwent discectomy, a mean diagnosis-to-operation period of 7.15 weeks, and used preoperative paracetamol (69.4%), cortisones (76.9%), and NSAIDs (59.7%). All PROMs showed significant early postoperative improvements (*p*-value < 0.001), where better NDI improvements are found among urban residents and congenital disease-related operations, better ODI improvement among tumor resection patients, without hormonal disorders or use of preoperative cortisones, while better EQ-VAS improvements found among patients who are younger, and did not use preoperative paracetamol or muscle relaxants, and better ESS improvement are shown among older patients (*p*-value < 0.05). The current study found an overall significant early postoperative improvement among spinal neurosurgery patients in PROMs of pain, QoL, sleep quality, and mental health. Several studies agree with the findings of the current study, with differences in the affecting factors related to sampling and population characteristics differences. Patient’s engagement in preoperative education, resource allocation, and conduct of multicenter, interventional studies is recommended.

**Supplementary Information:**

The online version contains supplementary material available at 10.1038/s41598-025-25850-3.

## Background

The National Institutes of Health (NIH) has established the term known as patient-reported outcomes (PROs) as a product of the transition from volume-based to value-based healthcare services, in which a Patient-Reported Outcomes Measurement Information System has been initiated. In such a system, the focus is on collecting crucial health information related to the patient’s history, assessment and postoperative phase that will help in enhancing the patient outcomes during and after hospitalization, which include, but are not limited to, comorbidities, mental health, social support, pre- and post-injury function, pain and quality of life (QoL)^1^, which helps in exploring strategies that may enhance the value of care that is provided to patients by improving health outcomes and reducing the cost of delivered care, which is done by developing valid and accurate measurements of the patients’ mental, physical and social health^2^.

Patient-reported outcomes measures (PROMs) consist of valid tools that intend to gather subjective information related to the patient’s condition during several phases of the surgical process, to build a comprehensive care plan and future short-term and long-term goals to improve surgical outcomes, with an increase in their use among neurosurgery patients^3^. Despite the abundance of tools that measure PROMs, studies have shown that there is an urgent need to develop disease-specific PROMs in the field of neurosurgery to address the satisfaction, safety, and different QoL and postoperative perspectives of the patients as the main specific outcomes related to neurosurgical procedures^4–6^. Several studies have shown that reporting PROs among spinal neurosurgery patients has demonstrated significant functional and psychological improvements, with decreased related disabilities^7^, while other studies have also focused on the cost-effectiveness and safety measures improvement in the hospital settings associated with such implementations^8^.

A systematic review has found that there are 31 unique PROs to assess at least one domain among neurosurgery patients, where 73% of the studies have focused on tools related to specific physical function disability, 55% on pain, and 32% on QoL. It is worth noting that few studies (5.7%) in the neurosurgery field utilize neurosurgery-specific PROMs^9^. In the neurospine surgical field, the most commonly used PROMs were the Scoliosis Research Society-22 (SRS-22), Short Form-12, Short Form-36 (SF-12 and SF-36), Ronald-Morris Disability Questionnaire (RMDQ), and Oswestry Disability Index (ODI). Recently, there has been a trend toward using disease-specific rather than generic tools, along with strong encouragement for using translated versions of validated tools across different settings and populations^10^.

Implementing PROMs effectively in healthcare services involves systematically integrating them into the hospital, outpatient clinic, or other healthcare institution. This process begins with defining the purpose, then moves to designing, preparing, and starting with a trial phase. It concludes with reflection, evaluation of the PROMs process, and making improvements^9^. Successful implementation of PROMs within health institutions requires ongoing and systematic identification of barriers, such as data collection burdens, healthcare providers’ skepticism about PROMs’ validity, and addressing concerns and issues related to their integration within specific healthcare service types^10,11^.

Also, the use of PROMs faces methodological issues, which are related to differences in data collection methods, as well as the nature of explanatory variables, which originate from differences between patient-reported and administrative data^9^. Other issues are concerned with the financial perspective, which are included in the need for support by experts, shared vision among healthcare specialists, patients and purchasers of healthcare services, targeting the sustainable implementation and improvement of healthcare quality^10^, in addition to financial issues across countries, like differences between low- and high-income courtiers, in the aspects of technological support, robust workflow and socioeconomic factors^11^.

The use of a longitudinal approach in PROMs for several surgical procedures was found to have an effective assessment of postoperative symptom recovery trajectories, resulting in more reflection of the effectiveness in recovery evaluation over time^12,13^. Also, the use of longitudinal methods of PROMs assessment was found to be beneficial in predicting the short- and long-term postoperative outcomes of surgeries, including pain and functional disability, using pre-surgical prediction algorithms, with the necessity to combine such longitudinal data with clinical factors to better understand patient outcomes^14,15^.

Studies have shown that the assessment and documentation of PROs among neurosurgery patients is both “under-utilized” and “under-standardized”^3^, which is manifested by the few number of neurosurgery-specific PROMs tools^16^ and the lack of robust evidence, yet high potential, of their use^17^. Moreover, the area of PROs among spinal neurosurgery patients in Palestine is under-covered in the scientific literature, which may be related to financial and staff-related issues^18^. Specific barriers related to this issue may include cultural beliefs and health perceptions of the Palestinian community^19^, language and communication barriers between patients and healthcare providers (HCPs) who mostly use medical jargon^20^, as well as the lack of validation of Arabic PROMs tools in the Palestinian context, in addition to the influence of healthcare expectations, social and family dynamics and trust in HCPs^21^.

The results of the current study will guide HCPs and decision-makers towards several interventions that aim to improve spinal neurosurgery patients’ physical and mental health outcomes, in term of increasing physicians and nurses’ awareness about the importance of covering the areas related to PROs among spinal neurosurgery patients, while decision-makers will be able to establish targeted guidelines related to interventions on enhancing the physical and mental health among them.

## Methods

### Study aim, design, and setting

The study aimed to investigate PROMs related to pain, QoL, sleep quality, and mental status, as well as the most common demographic and medical factors that are related to their pre-post changes, following spinal neurosurgery in Palestine, using a prospective, longitudinal, quantitative design, where the researcher collected data related to spinal neurosurgery patients’ PROMs before and one month after the surgical intervention, without the manipulation of the intervention itself. The used design allowed for the observation of the targeted variables (PROMs) in a temporal way^22^, to increase the statistical power related to the precision of estimated measures^23^, , and reduce recall bias^24^.

The study was conducted in the neurosurgery department of Palestine Medical Complex (PMC), a tertiary hospital that serves as the largest public referral hospital in Palestine, which serves a diverse patient population from various demographics, which improves the generalizability of the results.

### Study population, sample, and sampling

The population of the study included all spinal neurosurgery patients who were planned to have cervical or lumbar neurosurgery operations for the removal of discopathies or tumors, who were admitted and scheduled for operations during the study period. The researcher implemented the convenience sampling technique, where all eligible, available, and accessible patients were included in the data collection.

The sampling method interprets the imbalance in some patients’ characteristics, including surgical site and type, the medications used per-operatively (whether in exclusive or in various combination nature), mainly caused by patients being diagnosed and managed by different neurosurgeons, and tumor type (mostly benign), reflecting the natural epidemiology of primary spinal intradural-extramedullary tumors, where benign tumors like meningiomas and schwannomas are dominant.

The sample size was calculated using G*Power 3.1.9.6 software^25^, with an effect size of 0.518 (based on means and standard deviation (SD) of a previous literature), given the recommended sample size was 32, while researcher eventually recruited a total of 134 spinal neurosurgery patients.

### Eligibility criteria

**Inclusion criteria**: Adult patient between 18 and 65 years old who underwent spinal neurosurgery operation (e.g., herniated disc, spinal stenosis, degenerative spinal disease and tumors from the cervical and/or lumbar areas), and completed both pre-test and post-test PROMs questionnaires.

**Exclusion criteria**: Patients undergoing additional major surgeries or operations during the same admission, with severe cognitive or psychiatric conditions, or specific postoperative complications that required readmission during the one-month postoperative period.

### Data collection tool and process

Data were collected between 25th of January and 15th of August 2024 using a structured questionnaire that consisted of five main sections: demographic data, pain, QoL, sleep quality, and mental health. Demographic factors also included additional information included the type of surgery, waiting time, and the use of specific pain killers.

The Arabic valid tools were used for PROMs assessment, including the ODI for lower back pain assessment among lumbar spine neurosurgery patients^26^, Neck Disability Index (NDI) for the cervical spine pain^27^, the EuroQoL 5-Dimension 5-Level (EuroQoL-5D-5 L) for QoL, Pittsburgh Sleep Quality Index (PSQI)^28^ and the Epworth Sleepiness Scale (ESS)^29^ for sleep quality, and the Patient Health Questionnaire (PHQ-9)^30^ for mental status.

On the day of admission for each patient, the researcher collected the preoperative data in a specific Google Form that was prepared to include the mentioned sections, which was chosen for clinical relevance and accuracy of the collected data, in addition to standardization and consistency among all the targeted patients. One month after the operation, the researcher contacted the patients again via telephone and collected the postoperative PROMs data, using the same form.

As the study was constructed from a master’s thesis, the one-month follow-up period was limited by time constraints, therefore, determined by its scope and framework. Also, several previous studies on postoperative recovery and patient-reported outcomes after spinal surgeries have also employed short-term follow-up periods of 4 to 6 weeks^31–34^ to capture changes in pain, QoL and functional recovery.

### Data analysis

For the purpose of data analysis, Statistical Package for Social Sciences (SPSS) software version 25.0 was used to produce the descriptive and analytical results of the study’s data. Descriptive results included frequencies and percentages of patients’ demographics and statements of PROMs tools, as well as the mean and SD of scores. Analytical results included paired sample *t*-test for pre- and postoperative differences in PROMs scores, independent samples *t*-test and one-way ANOVA for mean differences across demographic factors, and Pearson Correlations, with a significance set at *p*-value < 0.05. Effect sizes were calculated to quantify the magnitude of change in PROMs, where *Cohen’s d* for *t*-test and eta-squared (η^2^) for ANOVA models were interpreted according to conventional benchmarks (small = 0.2/0.01, medium = 0.5/0.06, large = 0.8/0.14).

## Results

### Demographic data of the patients

Most of the patients who were recruited in the current study (70.1%) were in the later middle-aged adult group, with a mean age of 49.16 ± 6.06 years old, ranging from 32 to 66 years old. Around half of the sample were male patients (49.3%), with an approximate percentage of patients who live in rural areas (48.5%). Moreover, nearly half of the participants (45.5%) have a bachelor’s educational degree, while nearly three-fourths of them (74.6%) are married. In terms of professional life, 38.8% of the patients work in the private sector, compared to 23.9% who are self-employed, with 39.6% having between 1800 and 3000 ILS of monthly income, as shown in Table 1.

**Table 1:** Distribution of demographic data of the patients (*N* = 134).

Variable	Values	Frequency	Percentage
Age^a^	30–45 years old	33	24.6
	46–60 years old	94	70.1
	61–65 years old	7	5.2
	Mean ± SD^b^ (min–max)^c^	49.16 ± 6.06 (32–66)	
Gender	Male	66	49.3
	Female	68	50.7
Residency	City	52	38.8
	Village‎/town	65	48.5
	Camp	17	12.7
Education	Up to elementary school	20	14.9
	Up to high school	53	39.6
	University degree	61	45.5
Marital status	Single	20	14.9
	Married	100	74.6
	Other	14	10.4
Work status	Not working	26	19.4
	Working in governmental sector	24	17.9
	Working in private sector	52	38.8
	Self-employed	32	23.9
Monthly income^d^	< 1800 ILS	33	24.6
	1800–3000 ILS	53	39.6
	3001–5000 ILS	33	24.6
	> 5000 ILS	15	11.2

^a^Age groups were categorized based on standard life-stage classifications that were used in clinical and epidemiological literature, where patients between 30 and 45 years old are referred to younger middle-aged adults, between 46 and 60 years old as later middle-aged adults, and 61–65 years old as older adults. ^b^SD = Standard deviation. ^c^min = minimum value, and max = maximum value. ^d^Monthly income in ILS = Israeli New Shekel (currency).

In terms of the operation-related data, most of the patients (73.9%) underwent a lumbar spine neurosurgery approach, compared to 26.1% for the cervical approach. Of these patients, 59.7% underwent discectomy, while around one-third of them (29.9%) underwent tumor resection. The patients had a mean period between diagnosis and operation of 7.15 ± 4.68 weeks, with a mean weight of 80.31 ± 7.40 kg, ranging from 64 to 99 kg, while the mean height was 169.28 ± 7.51 centimeters, ranging from 150 to 185 centimeters, giving a mean Body Mass Index (BMI) of 28.17 ± 3.47 kg/m^2^, giving an overall overweight status, which ranged from 20.52 to 38.46 kg/m^2^. The most common comorbidities found among the patients were hypertension (29.9%), followed by diabetes mellitus (24.6%) and hormonal issues (14.9%). During the preoperative period, more than two thirds of the patients reported having paracetamol (69.4%), which was higher than NSAIDs (59.7%), for pain relief, while more than half of the patients have used muscle relaxants (54.5%), with a higher percentage of patients who reported consuming corticosteroids (76.9%), as shown in Table 2.

**Table 2:** Distribution of the patients’ characteristics related to health and operation.

Variable	Values	Frequency			Percentage	
Operation site	Cervical	35			26.1	
	Lumbar	99			73.9	
Operation type	Discectomy	80			59.7	
	Tumor resection	40			29.9	
	Congenital malformation	14			10.4	
Diagnosis to operation period (in weeks)	Mean ± SD^a^ (min–max)	7.15 ± 4.68 (1–7)				
Patient’s weight (kilograms)	Mean ± SD (min–max)	80.31 ± 7.40 (64–99)				
Patient’s height (centimeters)	Mean ± SD (min–max)	169.28 ± 7.51 (150–185)				
BMI^b^ (kg/m^2^)	Mean ± SD (min–max)	28.17 ± 3.47 (20.52–38.46)				
		Yes		No		
Comorbidities	HTN^c^	40	29.9%	94		70.1
	DM^d^	33	24.6%	101		75.4
	Hormonal issues	20	14.9%	114		85.1
	Others	13	9.7%	121		90.3
Preoperative medication consumption	NSAIDs^e^	80	59.7%	54		40.3
	Paracetamol	93	69.4%	41		30.6
	Muscle relaxants	73	54.5%	61		45.5
	Cortisones	103	76.9%	31		23.1
	Others	29	21.6%	105		78.4

^a^SD = Standard deviation, ^b^BMI = Body Mass Index, ^c^HTN, Hypertension, ^d^DM = Diabetes mellitus, ^e^NSAIDs = Non-steroidal anti-inflammatory drugs.

### Pain

For the patients who underwent cervical spine neurosurgery approach, the overall NDI score was calculated and categorized, where Table 3 showed a significant decrease in overall score from a mean of 53.847 ± 6.658 in the preoperative phase to a mean of 21.568 ± 5.283 in the postoperative phase, with a mean decrease by 32.279 (t = 26.943, *p*-value < 0.001), which was also reflected in the categories of NDI, where 85.7% of the patients had severe preoperative disability, which was significantly decreased to a percentage of 57.1% having moderate and 42.9% having minimal disabilities (t = 15.289, *p*-value < 0.001), indicating a significant improvement in neck pain after one month of the operation.

**Table 3:** Description of overall NDI scores and categories.

NDI^a^ category	Preoperative phase		Postoperative phase		Mean dif.	t^c^	*p*-value
	N^b^	%	N	%			
Minimal disability	0	0.0	15	42.9	1.571	15.289	< 0.001
Moderate disability	0	0.0	20	57.1			
Severe disability	30	85.7	0	0.0			
Crippling disability	5	14.3	0	0.0			
Bed-bound	0	0.0	0	0.0			
Overall mean ± SD^d^	53.847 ± 6.658		21.568 ± 5.283		32.279	26.943	< 0.001

^a^NDI = Neck disability index. ^b^N = Number (frequency), ^c^t = paired *t*-test value, ^d^SD = standard deviation.

Patients who underwent lumbar spine neurosurgery approach were evaluated for their pain using ODI, with their overall and categorization shown in Table 4, where the overall score showed a significant decrease from a preoperative mean of 58.929 ± 5.438 to a postoperative mean of 24.687 ± 5.629, with a mean decrease by 34.242 (t = 47.424, *p*-value < 0.001), indicating an overall improvement in lumbar area pain after one month of the operation. In more detail, 68.7% of the patients had severe disability category in the preoperative phase, compared to 71.7% having moderate and 27.3% having minimal disability categories in the postoperative phase (t = 25.803, *p*-value < 0.001).

**Table 4:** Description of overall ODI scores and categories.

ODI^a^ category	Preoperative phase		Postoperative phase		Mean dif.	t^c^	*p*-value
	N^b^	%	N	%			
Minimal disability	0	0.0	27	27.3	1.576	25.803	< 0.001
Moderate disability	0	0.0	71	71.7			
Severe disability	68	68.7	1	1.0			
Crippling disability	31	31.3	0	0.0			
Bed-bound	0	0.0	0	0.0			
Overall mean ± SD^d^	58.929 ± 5.438		24.687 ± 5.629		34.242	47.424	< 0.001

^a^ODI = Oswestry disability index. ^b^N = Number (frequency), ^c^t = paired *t*-test value, ^d^SD = standard deviation.

### Quality of life (QoL)

The quality of life (QoL) among the recruited patients was assessed using the five-dimensional EuroQoL tool, where patients’ responses and pre-post comparison are shown in Table 5. The domain of mobility showed a significant improvement, where 58.2% of the patients had a moderate problem in walking, compared to 53.0% having a slight problem in walking in the postoperative phase (t = 8.242, *p*-value < 0.001). The same pattern was witnessed in other domains. For example, the problem in self-care was moderate among 49.3% of the patients in the preoperative phase, while it was slight among 52.2% of them postoperatively (t = 6.797, *p*-value < 0.001), with 59.7% having severe preoperative problems in usual activities, compared to 63.4% having slight related problems (t = 16.930, *p*-value < 0.001). Also, problems related to pain or discomfort were severe among 73.9% of the patients in the preoperative phase, compared to slight among 56.7% of them in the postoperative phase (t = 19.497, *p*-value < 0.001). Lastly, anxiety issues were severe among 52.2% of the patients preoperatively, compared to 54.5% with slight and 40.3% with no related problems in the postoperative phase (t = 13.302, *p*-value < 0.001).

The overall VAS mean significantly improved from 21.642 ± 13.500 in the preoperative phase to a mean of 68.508 ± 22.158 in the postoperative phase, with a mean increase of 46.9 points (t = -28.720, *p*-value < 0.001). The utility score also showed a significant improvement from a mean of 0.0637 to 0.7012, with a mean improvement of 0.638 points (t = -20.753, *p*-value < 0.001).

**Table 5:** Distribution of patients’ responses and preoperative-postoperative differences of EQ-5D-5 L scale (N = 134).

Statements	Preoperative			Postoperative		Mean dif.	t^c^	*p*-value
	N^a^	%		N	%			
1. Mobility								
You have *no* problems in walking about?	18	13.4		46	34.3	0.776	8.242	< 0.001
You have *slight* problems in walking about?	29	21.6		71	53.0			
You have *moderate* problems in walking about?	78	58.2		13	9.7			
You have *severe* problems in walking about?	8	6.0		4	3.0			
You are *unable to* walk about?	1	0.7		0	0.0			
2. Self-care								
You have *no* problems washing or dressing yourself?	14	10.4		30	22.4	0.731	6.797	< 0.001
You have *slight* problems washing or dressing yourself?	29	21.6		70	52.2			
You have *moderate* problems washing or dressing yourself?	66	49.3		29	21.6			
You have *severe* problems washing or dressing yourself?	20	14.9		5	3.7			
You are *unable to* wash or dress yourself?	5	3.7		0	0.0			
3. Usual activities								
You have *no* problems doing your usual activities?	4	3.0		32	23.9	1.724	16.930	< 0.001
You have *slight* problems doing your usual activities?	14	10.4		85	63.4			
You have *moderate* problems doing your usual activities?	20	14.9		9	6.7			
You have *severe* problems doing your usual activities?	80	59.7		8	6.0			
You are *unable to* do your usual activities?	16	11.9		0	0.0			
4. Pain or discomfort								
You have *no* pain or discomfort?	0	0.0		17	12.7	1.612	19.497	< 0.001
You have *slight* pain or discomfort?	4	3.0		76	56.7			
You have *moderate* pain or discomfort?	21	15.7		32	23.9			
You have *severe* pain or discomfort?	99	73.9		9	6.7			
You have *extreme* pain or discomfort?	10	7.5		0	0.0			
5. Anxiety or depression								
You are *not* anxious or depressed?	16	11.9%		54	40.3	1.522	13.302	< 0.001
You are *slightly* anxious or depressed?	17	12.7%		73	54.5			
You are *moderately* anxious or depressed?	29	21.6%		5	3.7			
You are *severely* anxious or depressed?	70	52.2%		2	1.5			
You are *extremely* anxious or depressed?	2	1.5%		0	0.0			
ED-5D VAS^**d**^ (mean ± SD^b^)	21.642 ± 13.500			68.508 ± 22.158		46.9	− 8.720	< 0.001
Utility score (mean (min–max))	0.0637 (− 0.451 to 0.710)			0.7012 (0.298–0.948)		0.638	− 0.753	< 0.001

N = Number (frequency), SD = standard deviation, t = paired *t*-test value, VAS = visual analogue scale.

### Sleep quality

According to the scoring of the PSQI tool, the global PSQI score showed a significant decrease from a preoperative mean of 13.522 ± 1.995 to a postoperative mean of 7.866 ± 1.969, with a mean decrease of 5.657 points (t = 26.830, *p*-value < 0.001). In addition, all components of sleep quality showed significant improvements between the preoperative and the one-month postoperative phases, including sleep latency (2.687 ± 0.554 to 1.873 ± 0.896, respectively, t = 16.309, *p*-value < 0.001), sleep efficiency (1.403 ± 1.034 to 0.358 ± 0.481, respectively, t = 10.769, *p*-value < 0.001), and daytime dysfunction (1.903 ± 0.533 to 0.851 ± 0.569, respectively, t = 18.345, *p*-value < 0.001), except for sleep duration (1.388 ± 1.501 to 1.522 ± 1.505, respectively, t = -0.749, *p*-value = 0.455), as shown in Table 6.

**Table 6:** Differences in sleep quality component and PSQI score between preoperative and postoperative phases.

Component	Preoperative		Postoperative		Mean dif.	t^b^	*p*-value
	Mean	SD^a^	Mean	SD			
Subjective sleep quality	1.925	0.752	0.687	0.618	1.239	17.594	< 0.001
Sleep latency	2.687	0.554	1.873	0.896	0.814	16.309	< 0.001
Sleep duration	1.388	1.501	1.522	1.505	-0.134	-0.749	0.455
Sleep efficiency	1.403	1.034	0.358	0.481	1.045	10.769	< 0.001
Sleep disturbance	2.552	0.499	1.716	0.452	0.836	18.497	< 0.001
Use of sleep medication	1.664	0.822	0.858	0.727	0.806	12.597	< 0.001
Daytime dysfunction	1.903	0.533	0.851	0.569	1.052	18.345	< 0.001
Global PSQI^c^ score	13.522	1.995	7.866	1.969	5.657	26.830	< 0.001

^a^SD = Standard deviation, ^b^t = paired *t*-test values, ^c^PSQI = Pittsburg Sleep Quality Index.

The daytime sleepiness of the recruited patients was assessed using ESS, as shown in Table 7. All areas of sleepiness have witnessed significant improvements from the preoperative to one-month postoperative phases, where majority of the issues happened “mostly” in the preoperative phase, and turned to become rare or never happening in the postoperative phase, including sleepiness while sitting and reading (68.7% mostly vs. 52.2% never, respectively, t = 17.939, *p*-value < 0.001), as well as while watching TV (64.2% mostly vs. 48.5% never, respectively, t = 17.094, *p*-value < 0.001), and while lying down to rest (57.5% mostly vs. 50.7% rarely, respectively, t = 12.748, *p*-value < 0.001). This also applies for sleepiness while sitting after a lunch (57.5% mostly vs. 44.8% rarely, respectively, t = 11.654, *p*-value < 0.001).

Other differences were less significant, including sleepiness while being a passenger in a car (from 70.1% rarely to 61.9% never, respectively, t = 11.464, *p*-value < 0.001) and while sitting and talking (from 67.2% rarely to 64.2% never, respectively, t = 8.672, *p*-value < 0.001). Overall, the score of ESS significantly decreased from a preoperative mean of 11.425 ± 2.328 to a postoperative mean of 4.672 ± 1.867, with a mean decrease by 6.754 points (t = 37.539, *p*-value < 0.001), indicating an overall significant decrease in sleepiness in the one-month postoperative phase.

**Table 7:** Differences in daytime sleepiness using ESS between preoperative and postoperative phases.

Situation	Preoperative								Postoperative								Mean dif.	t*^b^
	Never		Rarely		Mostly		Always		Never		Rarely		Mostly		Always			
Sitting and reading	13	9.7%	16	11.9%	92	68.7%	13	9.7%	70	52.2%	51	38.1%	11	8.2%	2	1.5%	1.194	17.939
Watching TV	17	12.7%	13	9.7%	86	64.2%	18	13.4%	65	48.5%	59	44.0%	10	7.5%	0	0.0%	1.194	17.094
Inactive in public	33	24.6%	18	13.4%	73	54.5%	10	7.5%	69	51.5%	56	41.8%	9	6.7%	0	0.0%	0.896	12.851
As a car passenger	13	9.7%	94	70.1%	13	9.7%	14	10.4%	83	61.9%	51	38.1%	0	0.0%	0	0.0%	0.828	11.464
Lying down to rest	16	11.9%	33	24.6%	77	57.5%	8	6.0%	44	32.8%	68	50.7%	22	16.4%	0	0.0%	0.739	12.748
Sitting and talking	34	25.4%	90	67.2%	6	4.5%	4	3.0%	86	64.2%	48	35.8%	0	0.0%	0	0.0%	0.493	8.672
Sitting after lunch	18	13.4%	37	27.6%	77	57.5%	2	1.5%	50	37.3%	60	44.8%	24	17.9%	0	0.0%	0.664	11.654
Stopped in a car	22	16.4%	66	49.3%	29	21.6%	17	12.7%	72	53.7%	52	38.8%	7	5.2%	3	2.2%	0.746	10.538
ESS^a^ score	11.425 ± 2.328								4.672 ± 1.867								6.754	37.539

^*^All differences between preoperative and postoperative phases are significant at *p*-value < 0.001. ^a^ESS = Epworth Sleepiness Scale, ^b^t = paired *t*-test value.

### Mental health

The mental status of the patients was assessed using the PHQ-9 tool, where the overall score and categorization are explained in Table 8, which shows that the overall score significantly decreased from a preoperative mean of 12.00 ± 2.639 to a postoperative mean of 4.31 ± 1.890 (t = 37.650, *p*-value < 0.001), with 67.9% of the patients categorized as having moderate depression in the preoperative phase, compared to 54.1% of them having minimal, and 45.1% having mild, depression in the postoperative phase (t = 29.079, *p*-value < 0.001).

**Table 8:** Differences in mental status severity classifications and overall score between preoperative and postoperative phases.

Severity classification	Preoperative		Postoperative		t^a^	*p*-value
	*N*	%	*N*	%		
Minimal depression	0	0.0%	72	54.1%	29.079	< 0.001
Mild depression	18	13.4%	60	45.1%		
Moderate depression	91	67.9%	1	0.8%		
Moderately severe depression	25	18.7%	0	0.0%		
Severe depression	0	0.0%	0	0.0%		
Overall PHQ-9 score (mean ± SD^b^)	12.00 ± 2.639		4.31 ± 1.890		37.650	< 0.001

^a^t = Paired *t*-test value, ^b^SD = Standard deviation.

### Differences in proms improvement across patients’ characteristics

As shown in Table 9, which tested the significance of differences in preoperative-postoperative PROMs scores across the categories of patients’ demographic factors, the age of the spinal neurosurgery patients was significantly related with differences in both EQ-VAS and ESS scores, where the highest improvement in VAS scores the represent their QoL are significantly noticed among patients between 30 and 44 years old with a medium effect size (mean difference = 53.33, F = 2.365, η^2^ = 0.035) than older patients, with a significant, negative mild correlation between patients’ age and the mean difference of VAS after one month of the operation (*r* = -0.184, *p*-value < 0.05), which indicates and overall better improvement in spinal neurosurgery patients’ QoL among younger patients. Also, the daytime sleepiness significantly improved among older patients with a medium effect size, for example, among patients 60 years old and more (mean difference = -8.86) compared to those who are between 45 and 59 years old (mean difference = -6.50, F = 4.821, η^2^ = 0.069), without a significant correlation between age and improvement in daytime sleepiness.

The residency of the patients also significantly impacted the improvement in NDI scores with a small effect size, where the mean difference was − 35.26 among patients living in urban areas, compared to -30.22 among rural area residents and − 29.14 among residents of refugee camps (F = 2.987, Cohen’s d = 0.157, *p*-value < 0.05), which indicates a better improvement in NDI among urban area residents than others. On the other hand, none of the rest of the spinal neurosurgery patients’ demographic factors showed significant relationships with the improvements in postoperative PROMs (*p*-value > 0.05).

**Table 9:** Relationship between demographic factors and pre-post differences in proms scores among spinal neurosurgery patients.

Factors		PROMs^a^ differences (postoperative–preoperative scores)													
		NDI^b^		ODI^c^		Utility score		EQ-VAS^d^		PSQI^e^		ESS^f^		PHQ-9^g^	
		Mean	SD^j^	Mean	SD	Mean	SD	Mean	SD	Mean	SD	Mean	SD	Mean	SD
Age	30–44	− 28.80	3.78	− 34.79	7.86	0.65	0.29	53.33	24.07	− 7.61	2.90	− 7.03	1.94	− 7.61	2.90
	45–59	− 32.75	7.49	− 34.34	7.00	0.64	0.25	45.74	26.82	− 7.76	2.15	− 6.50	2.00	− 7.76	2.15
	≥60	− 36.00	.	− 30.67	5.89	0.55	0.25	31.43	19.52	− 7.14	2.61	− 8.86	2.67	− 7.14	2.61
	F^i^/η^2^	0.795/0.047		0.826/0.017		0.445/0.007		2.365*/0.035		0.241/0.004		4.821*/0.069		0.241/0.004	
	Correlation	r^k^ = − 0.082		*r* = 0.163		*r* = − 0.098		*r* = − 0.184*		*r* = − 0.023		*r* = − 0.037		*r* = − 0.023	
Gender	Male	− 30.18	4.13	− 35.14	7.00	0.62	0.27	47.42	27.08	− 7.86	2.39	− 6.64	1.89	− 7.86	2.39
	Female	− 33.86	8.44	− 33.29	7.33	0.65	0.24	46.32	25.39	− 7.51	2.35	− 6.87	2.26	− 7.51	2.35
	t^h^/Cohen’s d	1.696/0.529		− 1.280/− 0.258		− 0.692/− 0.119		0.243/0.042		− 0.854/− 0.147		0.641/0.111		− 0.854/− 0.147	
Residency	City	− 35.26	6.38	− 34.22	6.64	0.63	0.26	50.19	25.24	− 7.44	2.08	− 6.98	2.02	− 7.44	2.08
	Rural	− 30.22	6.51	− 34.56	7.70	0.65	0.26	45.54	26.52	− 7.83	2.52	− 6.62	2.25	− 7.83	2.52
	Camp	− 29.14	7.60	− 32.44	6.54	0.60	0.22	41.76	27.67	− 7.88	2.62	− 6.59	1.58	− 7.88	2.62
	F^i^/η^2^	2.978*/0.157		0.329/0.007		0.357/0.005		0.826/0.012		0.453/0.007		0.502/0.008		0.453/0.007	
Education	Elementary	− 35.31	9.22	− 33.40	6.33	0.64	0.27	52.50	24.25	− 8.35	2.98	− 6.65	2.06	− 8.35	2.98
	High school	− 30.98	5.85	− 33.08	7.21	0.61	0.24	45.85	27.83	− 7.17	2.26	− 6.77	2.29	− 7.17	2.26
	University	− 31.17	6.08	− 35.32	7.29	0.66	0.27	45.90	25.39	− 7.92	2.17	− 6.77	1.93	− 7.92	2.17
	F^i^/η^2^	1.306/0.075		1.148/0.023		0.528/0.008		0.542/0.008		2.397/0.035		0.029/0.000		2.397/0.035	
Marital status	Single	− 35.11	4.29	− 32.00	7.25	0.66	0.28	47.00	24.94	− 7.45	2.35	− 6.40	1.39	− 7.45	2.35
	Married	− 32.02	7.59	− 34.51	7.34	0.65	0.25	47.40	25.49	− 7.62	2.44	− 6.81	2.25	− 7.62	2.44
	Others	− 32.00	4.00	− 36.00	5.66	0.52	0.24	42.86	33.38	− 8.50	1.74	− 6.86	1.66	− 8.50	1.74
	F^i^/η^2^	0.250/0.015		1.211/0.025		1.585/0.024		0.183/0.003		0.969/0.015		0.339/0.005		0.969/0.015	
Work	None	− 34.08	8.21	− 30.89	7.80	0.57	0.23	48.08	25.46	− 7.58	2.98	− 6.69	2.24	− 7.58	2.98
	Governmental	− 36.70	6.92	− 33.78	7.16	0.59	0.30	47.50	26.74	− 7.83	2.14	− 6.92	2.12	− 7.83	2.14
	Private	− 29.08	4.93	− 36.11	7.11	0.69	0.22	48.27	26.70	− 7.46	2.12	− 6.56	2.14	− 7.46	2.12
	Self-employed	− 34.31	8.71	− 34.30	6.37	0.65	0.29	43.13	26.20	− 8.03	2.40	− 7.00	1.88	− 8.03	2.40
	F^i^/η^2^	2.452/0.192		2.225/0.066		1.783/0.040		0.287/0.007		0.428/0.010		0.354/0.008		0.428/0.010	
Income	< 1800	− 28.06	4.84	− 33.00	8.53	0.63	0.28	47.88	27.24	− 7.42	2.96	− 6.88	2.09	− 7.42	2.96
	1800–3000	− 31.54	6.87	− 33.49	5.50	0.62	0.26	45.85	26.27	− 8.28	1.97	− 6.40	1.88	− 8.28	1.97
	3001–5000	− 36.94	7.48	− 36.32	7.45	0.63	0.24	48.18	27.44	− 7.48	2.32	− 7.30	2.53	− 7.48	2.32
	> 5000	− 35.00	7.07	− 34.77	7.68	0.72	0.24	45.33	22.32	− 6.60	1.84	− 6.53	1.46	− 6.60	1.84
	F^i^/η^2^	2.411/0.189		1.113/0.034		0.549/0.013		0.086/0.002		2.478/0.054		1.394/0.031		2.478/0.054	

^*^Significant difference in improvement at *p*-value < 0.05. ^a^PROMs = Patient-reported outcomes measures, ^b^NDI = Neck Disability Index, ^c^ODI = Oswestry Disability Index, ^d^EQ-VAS = EuroQoL Visual Analogue Scale, ^e^PSQI = Pittsburg Sleep Quality Index, ^f^ESS = Epworth Sleepiness Scale, ^g^PHQ-9 = Patient Health Questionnaire, ^h^t = independent samples *t*-test value, ^i^F = one-way ANOVA test value, ^j^SD = standard deviation. ^k^r = Pearson Correlation Coefficient.

When the medical and health-related factors of spinal neurosurgery patients were tested in having significant relationships with the improvement in postoperative PROMs, more factors showed significant results, as shown in Table 10. For example, there was a significant improvement in NDI scores with a moderate effect size among spinal neurosurgery patients who underwent congenital diseases-related surgeries (mean difference = -36.67) compared to discectomy (mean difference = -33.04) and tumor resection (mean difference = -29.64) approaches, indicating an overall higher improvement in NDI among patients with congenital diseases (F = 2.689, η^2^^2^ = 0.071, *p*-value < 0.05). Also, differences in ODI scores showed significant differences with a moderate effect size across types of surgeries, where tumor resection patients (mean difference = -36.67) showed better improvement than patients of discectomy (mean difference = -33.05) and congenital diseases (mean difference = -33.83), indicating an overall better improvement in back pain index among patients with tumor-related spinal neurosurgical disorders (F = 2.951, η^2^ = 0.051, *p*-value < 0.05).

Moreover, patients without hormonal disorders showed more significant improvement with a moderate effect size in ODI scores (mean difference = -34.99) than patients with related disorders (mean difference = -30.38), indicating a better improvement in back pain index among patients who do not present with preoperative hormonal disorders (t = 2.409, Cohen’s d = 0.658, *p*-value < 0.05). Also, patients who reported using preoperative paracetamol for pain management showed less improvement in EQ-VAS scores (mean difference = 43.76) compared to patients who consumed them preoperatively (mean difference = 53.9), which applies for the use of muscle relaxants (mean difference = 41.78 vs. 52.95, respectively), indicating and overall less improvement in postoperative QoL VAS scores with moderate effect sizes among patients who consumed preoperative paracetamol (t = -2.095, Cohen’s d = -0.393) or muscle relaxants (t = -2.512, Cohen’s d = -0.436). In terms of the ODI scores, patients who reported using preoperative cortisones significantly showed less improvement (mean difference = -33.09) than who did not use them (mean difference = -37.83) with a moderate effect size, indicating an overall less improvement in back pain index among patients who reported preoperative cortisones (t = 2.919, Cohen’ d = 0.685, *p*-value < 0.05). On the other hand, the rest of the medical and health-related factors did not significantly affect the improvement in postoperative PROMs among spinal neurosurgery patients (*p*-value > 0.05).

**Table 10:** Relationship between patients’ health and operation-related factors and pre-post differences in proms scores among spinal neurosurgery patients.

Factors		PROMs^b^ differences (postoperative–preoperative scores)													
		NDI^b^		ODI^c^		Utility score		EQ-VAS^d^		PSQI^e^		ESS^f^		PHQ-9^g^	
		Mean	SD^j^	Mean	SD	Mean	SD	Mean	SD	Mean	SD	Mean	SD	Mean	SD
Site	Cervical	− 32.28	7.09	.	.	0.68	0.28	48.86	25.29	− 7.71	2.66	− 6.57	2.17	− 7.71	2.66
	Lumbar	.	.	− 34.24	7.18	0.62	0.25	46.16	26.52	− 7.68	2.26	− 6.82	2.06	− 7.68	2.26
	t^h^/Cohen’s d	–		–		1.018/0.200		0.523/0.103		− 0.080/− 0.016		0.601/0.118		− 0.080/− 0.016	
Type	Discectomy	− 33.04	7.04	− 33.05	6.54	0.65	0.26	46.37	27.11	− 7.58	2.14	− 6.79	2.01	− 7.58	2.14
	Tumor	− 29.64	6.94	− 36.67	7.32	0.61	0.27	44.25	24.80	− 7.83	2.33	− 6.67	2.15	− 7.83	2.33
	Congenital	− 36.67	7.86	− 33.83	8.80	0.65	0.19	57.14	23.35	− 7.93	3.58	− 6.79	2.42	− 7.93	3.58
	F^i^/η^2^	2.689*/0.071		2.951*/0.051		0.225/0.003		1.302/0.019		0.228/0.003		0.040/0.001		0.228/0.003	
HTN^l^	Yes	− 33.88	7.76	− 33.86	7.35	0.59	0.29	47.25	25.32	− 7.53	2.49	− 6.83	2.21	− 7.53	2.49
	No	− 31.55	6.80	− 34.40	7.16	0.66	0.24	46.70	26.62	− 7.76	2.32	− 6.72	2.04	− 7.76	2.32
	t^h^/Cohen’s d	− 0.901/− 0.328		0.338/0.075		− 1.206/− 0.246		0.111/0.021		0.515/0.097		− 0.257 to 0.049		0.515/0.097	
DM^m^	Yes	− 29.64	6.33	− 35.28	7.87	0.66	0.23	50.91	27.99	− 7.48	2.22	− 7.12	2.45	− 7.48	2.22
	No	− 33.06	7.22	− 33.89	6.96	0.63	0.27	45.54	25.51	− 7.75	2.41	− 6.63	1.95	− 7.75	2.41
	t^h^/Cohen’s d	1.208/0.486		− 0.834/− 0.193		0.577/0.116		1.024/0.205		0.563/0.113		− 1.042/− 0.234		0.563/0.113	
Hormonal	Yes	− 29.44	8.78	− 30.38	8.07	0.60	0.26	40.50	23.28	− 8.10	2.36	− 6.30	1.89	− 8.10	2.36
	No	− 32.64	6.93	− 34.99	6.80	0.64	0.26	47.98	26.55	− 7.61	2.37	− 6.83	2.11	− 7.61	2.37
	t^h^/Cohen’s d	0.847/0.450		2.409*/0.658		− 0.701/− 0.170		− 1.182/− 0.287		− 0.847/− 0.205		1.057/0.256		− 0.847/− 0.205	
Other comorbidities	Yes	− 30.67	4.75	− 37.75	9.10	0.55	0.30	52.31	27.74	− 7.46	1.94	− 5.85	1.86	− 7.46	1.94
	No	− 32.55	7.43	− 33.93	6.97	0.65	0.25	46.28	26.02	− 7.71	2.41	− 6.85	2.09	− 7.71	2.41
	t^h^/Cohen’s d	0.544/0.263		− 1.448/− 0.534		− 1.252/− 0.365		0.789/0.230		0.360/0.105		1.664/0.486		0.360/0.105	
NSAIDs^n^	Yes	− 30.69	6.66	− 33.86	6.55	0.65	0.23	47.25	26.86	− 7.70	2.39	− 6.91	2.23	− 7.70	2.39
	No	− 34.67	7.27	− 34.80	8.09	0.63	0.29	46.30	25.28	− 7.67	2.35	− 6.52	1.83	− 7.67	2.35
	t^h^/Cohen’s d	1.669/0.576		0.634/0.130		0.441/0.078		0.206/0.036		− 0.080/− 0.014		− 1.075/− 0.189		− 0.080/− 0.014	
Paracetamol	Yes	− 32.25	6.22	− 33.88	6.79	0.64	0.24	43.76	25.87	− 7.78	2.42	− 6.71	1.97	− 7.78	2.42
	No	− 32.38	10.52	− 34.94	7.94	0.64	0.30	53.90	25.68	− 7.46	2.25	− 6.85	2.34	− 7.46	2.25
	t^h^/Cohen’s d	0.042/0.018		0.698/0.148		− 0.073/− 0.014		− 2.095*/− 0.393		− 0.724/− 0.136		0.368/0.069		− 0.724/− 0.136	
Muscle relaxants	Yes	− 32.12	7.51	− 34.44	7.13	0.67	0.26	41.78	26.68	− 8.01	2.34	− 6.67	2.24	− 8.01	2.34
	No	− 32.44	6.83	− 34.00	7.33	0.60	0.25	52.95	24.31	− 7.30	2.35	− 6.85	1.90	− 7.30	2.35
	t^h^/Cohen’s d	0.132/0.045		− 0.299/− 0.060		1.636/0.284		− 2.512*/− 0.436		− 1.767/− 0.306		0.500/0.087		− 1.767/− 0.306	
Cortisones	Yes	− 32.71	7.48	− 33.09	6.74	0.66	0.25	47.38	26.90	− 7.60	2.37	− 6.80	2.10	− 7.60	2.37
	No	− 30.57	5.33	− 37.83	7.48	0.57	0.26	45.16	23.79	− 7.97	2.36	− 6.61	2.04	− 7.97	2.36
	t^h^/Cohen’s d	− 0.708/− 0.299		2.919*/0.685		1.700/0.348		0.413/0.085		0.754/0.155		− 0.428/− 0.088		0.754/0.155	
Other meds.	Yes	− 32.07	8.54	− 33.58	5.80	0.60	0.24	41.72	28.04	− 7.48	2.25	− 6.48	1.90	− 7.48	2.25
	No	− 32.36	6.62	− 34.40	7.50	0.65	0.26	48.29	25.55	− 7.74	2.40	− 6.83	2.13	− 7.74	2.40
	t^h^/Cohen’s d	0.110/0.041		0.446/0.114		− 0.842/− 0.177		− 1.199/− 0.251		0.523/0.110		0.790/0.166		0.523/0.110	
Period till op.	Correlation	r^k^ = 0.273		*r* = − 0.071		*r* = − 0.017		*r* = − 0.093		*r* = 0.021		*r* = 0.162		*r* = 0.021	
Weight	Correlation	*r* = − 0.058		*r* = 0.038		*r* = − 0.097		*r* = 0.127		*r* = 0.094		*r* = − 0.003		*r* = 0.094	
Height	Correlation	*r* = 0.023		*r* = − 0.077		*r* = 0.082		*r* = 0.119		*r* = 0.140		*r* = 0.031		*r* = 0.140	
BMI^o^	Correlation	*r* = − 0.058		*r* = 0.100		*r* = − 0.123		*r* = 0.008		*r* = − 0.041		*r* = − 0.031		*r* = − 0.041	

^*^Significant difference in improvement at *p*-value < 0.05. ^a^PROMs = Patient-reported outcomes measures, ^b^NDI = Neck Disability Index, ^c^ODI = Oswestry Disability Index, ^d^EQ-VAS = EuroQoL Visual Analogue Scale, ^e^PSQI = Pittsburg Sleep Quality Index, ^f^ESS = Epworth Sleepiness Scale, ^g^PHQ-9 = Patient Health Questionnaire, ^h^t = independent samples *t*-test value, ^i^F = one-way ANOVA test value, ^j^SD = standard deviation. ^k^r = Pearson Correlation Coefficient, ^l^HTN = Hypertension, ^m^DM = Diabetes Mellitus, ^n^NSAIDs = non-steroidal anti-inflammatory drugs, ^o^BMI = Body Mass Index.

## Discussion

The current study is the first study in Palestine to evaluate the early postoperative changes in multiple PROMs among spinal neurosurgery patients (health-related quality of life (HRQoL), sleep quality and mental health). All PROMs showed statistically significant improvements at one month following the surgery, which provides crucial insights into the multidimensional nature of early recovery, as well as specific concordance and discrepancy with prior literature.

Focusing on areas related to disability and functional recovery, the current study showed significant disability decrease, as measured by both ODI and NDI tools, which is consistent with previous studies^35–37^. The baseline ODI mean score was notably higher among our patients, which may indicate a greater preoperative disability burden among Palestinian patients, mostly related to late presentation and differences in access to care. The sharper decline in ODI scores may also suggest notable functional gains and rapid recovery within just one month postoperatively following decompression or tumor resection. On the other hand, early improvements should be interpreted with caution, as other previous literature shows continued improvements beyond three to six months^38^, which may indicate a partial rather than full recovery among the patients of the current study. Also, the used functional disability scales are limited to physical functions only, and may not fully reflect emotional or social picture.

Significant improvement in back pain were also observed among patients who underwent tumor resection compared to other types, which is similar to the findings of Chouhdari and colleagues^39^. Focusing on tumor part, it is worth mentioning that the sample of the study predominantly consisted of patients with benign tumor types, which is similar to the epidemiological findings of previous studies^40–42^, like schwannomas and meningiomas. Significant improvements in PROMs after resection of such benign intradural-extramedullary tumors was also supported by findings of previous studies^43–45^. It is recommended for future research to use domain-specific tools, as VAS is less granular in isolating pain as a domain.

Moving to HRQoL, the current study showed significant improvements the overall QoL and subjective health perception postoperative, using EQ-5D and EQ-VAS. Our study showed more profound change in the utility scores in comparison with the previous study of Hey and colleagues^35^, reflecting a possibly lower baseline QoL among Palestinian population, mostly related to socioeconomic hardship, late presentation and limited multidisciplinary preoperative care access. Although, the exclusion of patients with postoperative complications may have skewed outcomes toward more favorable results.

While previous studies^46,47^ utilized PSQI foe sleep quality to find that spinal pathology patients suffer from sleep disturbances, few of them have conducted pre-post analyses. The findings of our study support the hypothesis related to getting better sleep when pain and psychological distress are reduced. While the used sleep quality tools are validated, they remain subjective and may not capture the complexity of neurophysiological sleep disorders, with the presence of other factors, such as social support and communal living, especially in a conflict-affected country as in Palestine.

Mental health outcomes have shown significant postoperative improvement, as assessed by PHQ-9, which is consistent with literature that linked physical recovery to psychological well-being^48,49^. A specific preoperative finding is related to that 57.5% of the patients have denied thoughts of self-harm, which supports the preventive role of cultural and religious stigma surrounding suicide^50^.

The cultural norms may have influenced how depressive symptoms are perceived and reported, although the valid Arabic version was used, such as somatic expression of distress, that are often emphasized over emotional symptoms among Arab societies, leading to psychological complaints being underreported. Therefore, future research should consider supplementing standardized scales with culturally adapted qualitative assessment, and use mixed-methods approaches, to ensure a more comprehensive evaluation of depression among Palestinian patients.

### Limitations

The current study’s strength mainly includes the use of validated Arabic PROMs and a prospective design, while several limitations should be considered, like the use of observational, single-center design, with the absence of a control group and multivariate analyses that limits causal inference, in addition to the short one-month follow-up that restricted evaluation of long-term outcomes. Also, the exclusion of complicated cases may have limited generalizability of results. Lastly, the geopolitical and logistical constraints have affected recruitment feasibility, though the study hospital serves a population with diverse demographics. These findings are considered as a foundation for future research that is recommended to be conducted in multicenter, long-term and controlled approach.

### Implications

Despite the mentioned limitations, the value of conducting health outcomes research in such underrepresented populations is worth being highlighted, contributing important data on having achievable substantial PROMs improvements even within constrained systems. The findings of the current study encourage patients on education management through counselling on expected recovery timelines and influencing factors, necessity of social support and postoperative adherence. Among healthcare providers, it is recommended to utilize pre- and post-operative care through holistic assessment, and individualized care plans by recognizing variability in recovery based on corresponding factors.

Lastly, policymakers should struggle to provide equity in access to neurosurgical and rehabilitation services, especially among disadvantaged populations, in addition to the use of standardized PROMs in guidelines to support quality monitoring and cross-facility benchmarking. Multicenter studies and randomized controlled trials (RCTs) that include private and governmental sectors are recommended to generate context-specific and evidence-based practices in current settings. Moreover, outcomes are recommended to be studied over longer, multiple follow-up periods.

## Conclusions

The current prospective study was conducted on convenience sample of 134 lumbar and cervical spine neurosurgery patients to assess differences in pre- and post-operative PROMs related to pain, QoL, sleep quality and mental health using Arabic valid tools, which all showed significant early improvement at the one-month postoperative time point. Some patients’ factors have independently contributed to significant differences in postoperative improvements.

Several studies agreed with the overall improvements in postoperative PROMs among spinal neurosurgery patients, with some differences related to the used tools, sample characteristics and population-related factors, which calls for the importance of personalized care approaches.

Future research in Palestine should strengthen the generalizability and better assess the durability of outcomes by including larger, multi-center, controlled samples and longer follow-up periods, with the conduction of multivariate analyses. Using culturally sensitive assessment tools will also strengthen causal interpretation and provide more comprehension into recovery after spinal neurosurgeries.

## Supplementary Information

Below is the link to the electronic supplementary material.

Supplementary Material 1

Supplementary Material 2

## Data Availability

The datasets used and/or analyzed during the current study are available from the corresponding author on reasonable request. Additional supporting materials, including the institutional approval letters, supplementary figures and charts, and the approval from the Palestinian Ministry of Health, are available in the Supplementary Material. All requests relating to data should be addressed to [ahmadaadaqqa@gmail.com](mailto: ahmadaadaqqa@gmail.com) .

## Abbreviations

ACDF: Anterior Cervical Discectomy and Fusion
ADLs: Activities of Daily Living
ANOVA: Analysis of Variance
BMI: Body Mass Index
EQ-5D-5L: EuroQoL-5 Dimension-5 Level
ESS: Epworth Sleepiness Scale
HCPs: Healthcare Providers
HRQoL: Health-Related Quality of Life
ILS: Israeli Shekel (Currency)
IRB: Institutional Review Board
NDI: Neck Disability Index
NIH: National Institute of Health
NSAIDs: Non-Steroidal Anti-Inflammatory Drugs
ODI: Oswestry Disability Index
PHQ-9: Patient Health Questionnaire
PMC: Palestine Medical Complex
PROMs: Patient-Reported Outcomes Measures
PROs: Patient-Reported Outcomes
PSQI: Pittsburg Sleep Quality Index
QoL: Quality of Life
RCT: Randomized Controlled Trial
RMDQ: Ronald-Morris Disability Questionnaire
SD: Standard Deviation
SPSS: Statistical Package for Social Sciences
SRS: Scoliosis Research Society
VAS: Visual Analogue Scale
