# Supplementary figures and images for: Changes in pain, quality of life, sleep, and mental health after uncomplicated spinal neurosurgery in palestine: a prospective study of patient-reported outcomes
Source: Sci Rep. 2025 Nov 21;15:41841. doi: 10.1038/s41598-025-25850-3 (PMC12647860; doi:10.1038/s41598-025-25850-3)

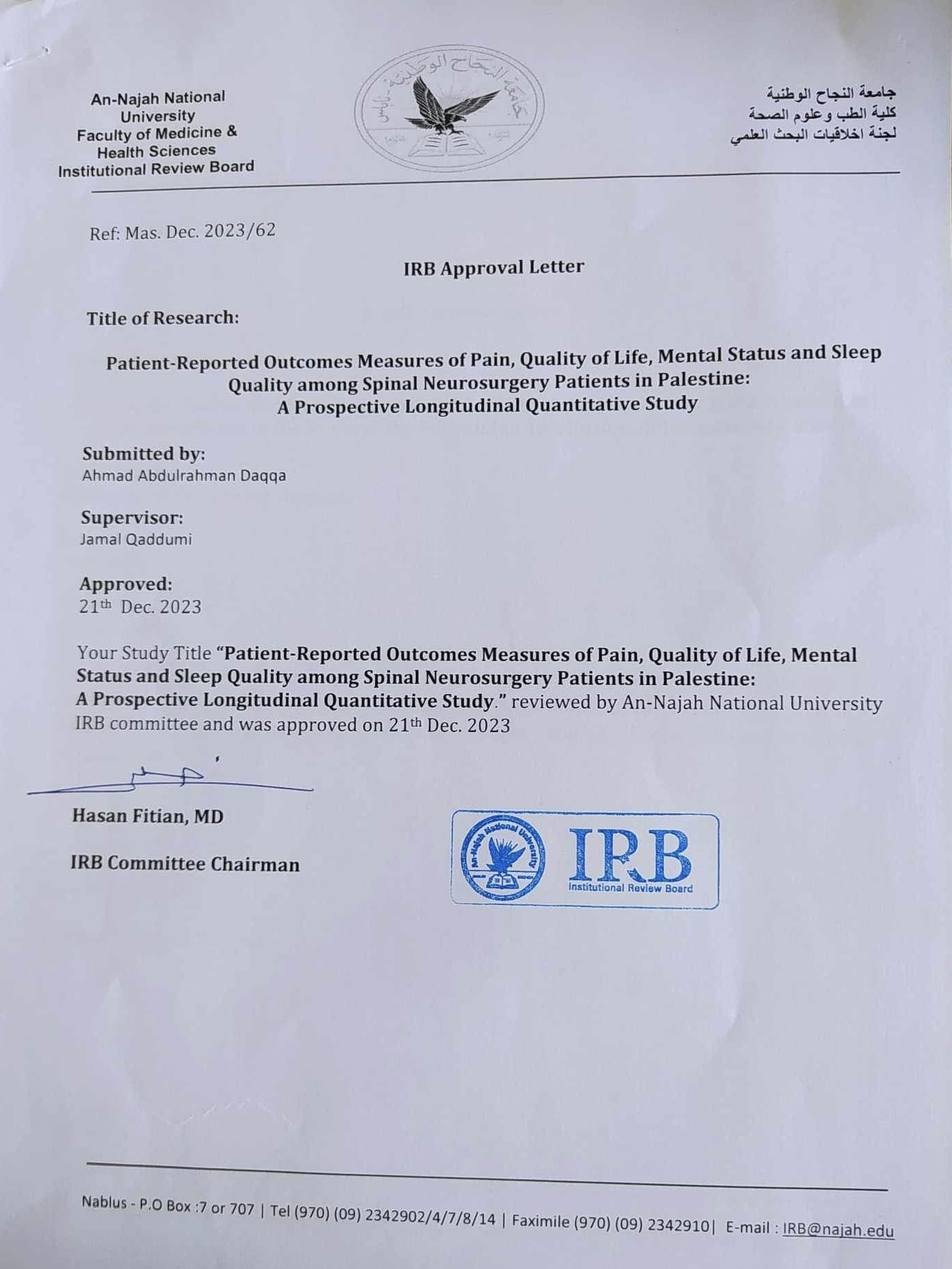

Supplement: Supplementary file 1 — Supplementary Material 1 [file 41598_2025_25850_MOESM1_ESM.jpg]
