# Supplementary material for: Changes in pain, quality of life, sleep, and mental health after uncomplicated spinal neurosurgery in palestine: a prospective study of patient-reported outcomes
Source: Sci Rep. 2025 Nov 21;15:41841. doi: 10.1038/s41598-025-25850-3 (PMC12647860; doi:10.1038/s41598-025-25850-3)
Supplement: Supplementary file 2 — Supplementary Material 2 [file 41598_2025_25850_MOESM2_ESM.docx]

## **Appendix A: Supplementary Charts and Figures**


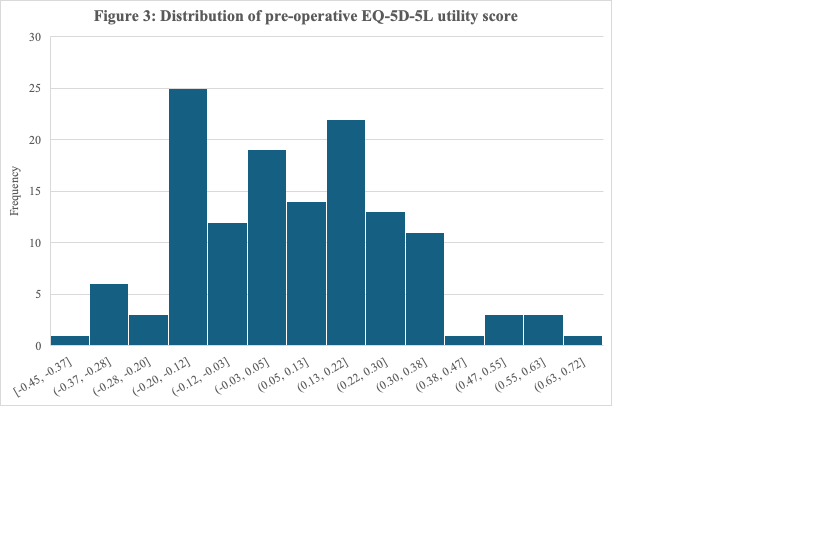


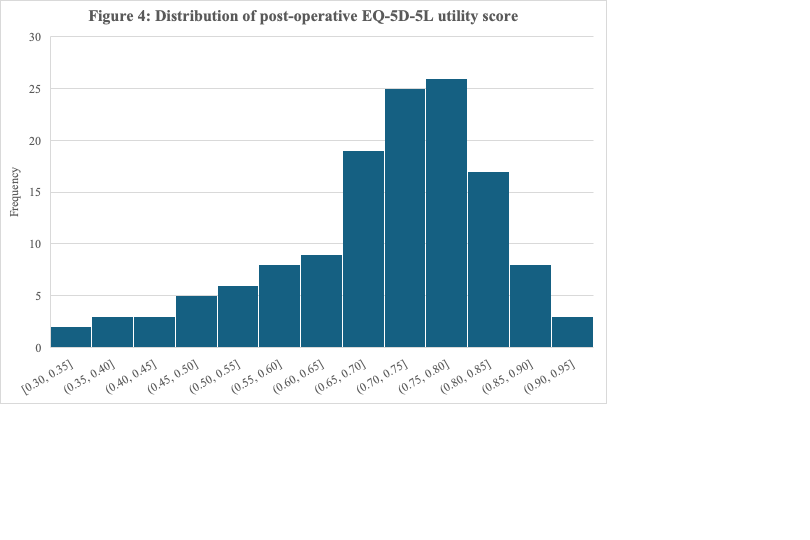


## **Appendix B: Institutional Review Board (IRB) approval**


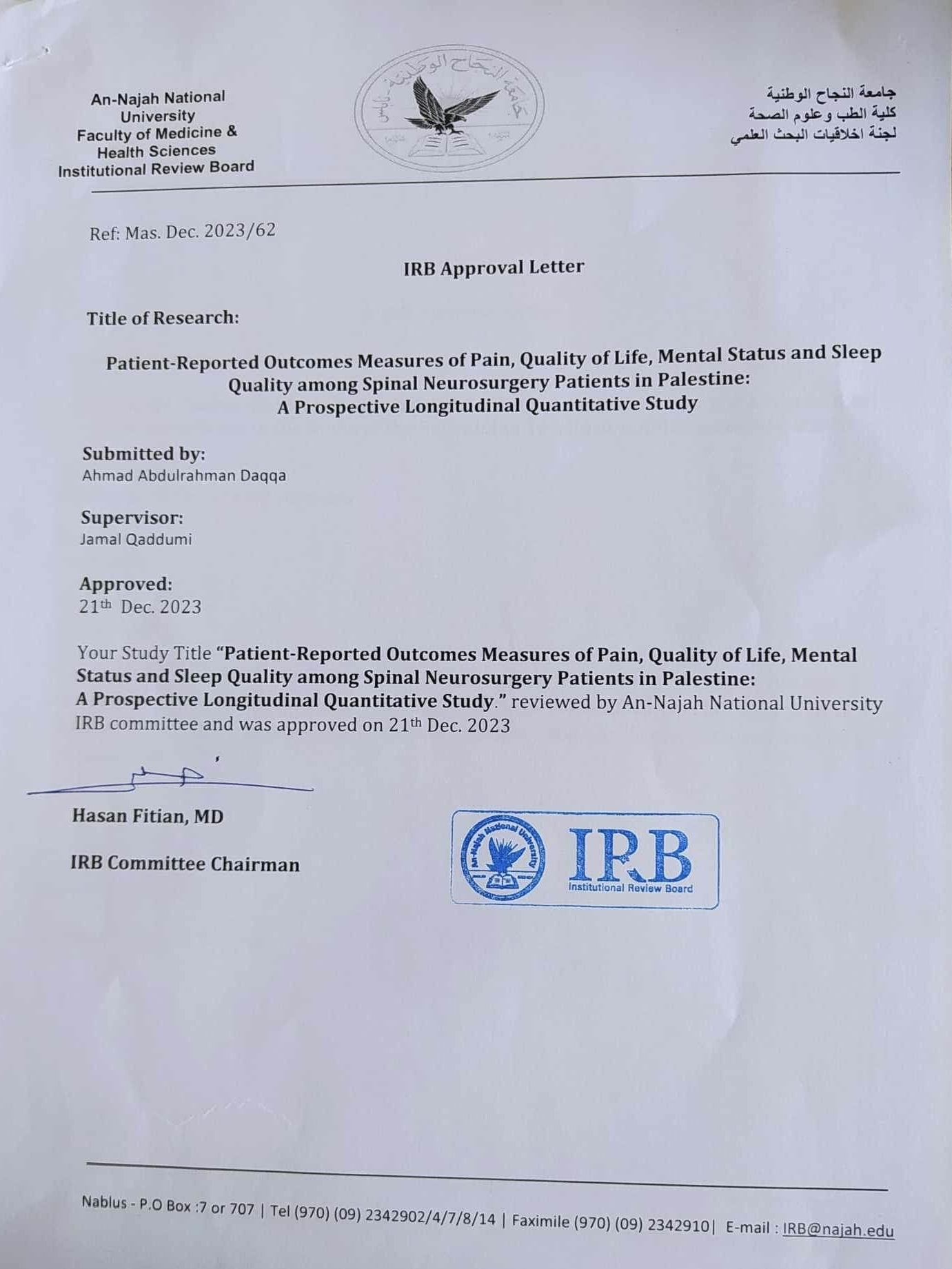


## **Appendix C: Approval from Ministry of Health (facilitation letter)**
